# Efficient applications of green synthesized CeO_2_/Bi_2_O_3_ nanocomposite for simultaneous electrochemical determination of Pb (II) and Cd (II) in real samples

**DOI:** 10.1038/s41598-025-10957-4

**Published:** 2025-08-07

**Authors:** Hadi Mahmoudi-Moghaddam, Sahar Zinatloo-Ajabshir, Saeid Ahmadzadeh, Ali Azari

**Affiliations:** 1 Pharmaceutics Research Center, Institute of Neuropharmacology, Kerman University of Medical Sciences, Kerman, Iran; 2 Department of Chemical Engineering, University of Bonab, P.O. Box. 5551395133, Bonab, Iran; 3 Research Center for Environment Pollutants, Qom University of Medical Sciences, Qom, Iran; 4 Environmental Health Engineering, School of Public Health, Qom University of Medical Sciences, Qom, Iran

**Keywords:** Nanocomposite, Modified electrode, Pb (II), Cd (II), Sensor, Materials science, Nanoscience and technology

## Abstract

In this study, Bi_2_O_3_/CeO_2_ nanocomposite was synthesized using serine, which played a dual role by promoting uniform particle morphology and aiding combustion during synthesis, resulting in a highly porous nanostructure. The produced nanocomposite was applied as a highly efficient modifier for screen-printed electrode (Bi_2_O_3_/CeO_2_/SPE), facilitating the simultaneous quantification of Pb(II) and Cd(II) using square wave anodic stripping voltammetry (SWASV). Compared to conventional sensors, the proposed electrode exhibited significantly enhanced electrochemical behavior, attributed to the synergistic structural and electrical properties of CeO₂ and Bi₂O₃ as well as an increased surface area. The sensor demonstrated a reliable response and effective peak separation at optimal parameters. The current signals exhibited linearity within the concentration range between 0.5 and 85 µg/L for both ions, achieving the limit of detection (LOD) of 0.09 µg/L for Pb(II) and 0.14 µg/L for Cd(II). The Bi_2_O_3_/CeO_2_/SPE was effectively utilized to detect cadmium and lead ions in water and food samples, demonstrating high recovery values across different spiked samples, and the outcomes closely matched those obtained through standard ICP analysis.

## Introduction

Heavy metals significantly threaten ecosystems and living organisms due to their high toxicity, tendency to bioaccumulate, and persistence in the environment. Consequently, monitoring heavy metal ions in drinking water and food is essential for safeguarding public health and protecting the environment^1,2^. These metals impact human health by entering the bloodstream and affecting organs, where they interact with functional groups in DNA, proteins, and enzymes, causing toxicity and poisoning^3^. Lead and cadmium are especially hazardous, as even minimal exposure can adversely affect aquatic life and human health. Lead primarily affects the central nervous system, kidneys, and blood, potentially resulting in death with high exposure levels, while cadmium can lead to renal damage, osteoporosis, and possibly renal cancer, with chronic low-level exposure resulting in bone diseases such as Itai-itai disease^4–6^.

The necessity for monitoring heavy metal concentrations has led to various analytical techniques, including inductively coupled plasma mass spectrometry (ICP), atomic absorption spectrometry (AAS), as well as ion chromatography (IC), and potentiometry. However, these methods often have limitations such as high equipment costs, complex procedures, and long analysis times^7–9^.

Electroanalytical techniques offer a promising alternative for detecting different analytes, with advantages like simplicity, high sensitivity, cost-effectiveness, and faster analysis^10,11^. Anodic stripping voltammetry (ASV) is particularly effective for detecting heavy metals, thanks to its high sensitivity and low detection limit. When combined with specific electrode materials, ASV techniques like DPASV and SWASV are powerful for the on-site determination of multiple metals^12–15^. Today, nanomaterials are widely employed in the development of various types of electrodes due to their unique physical and chemical properties^16,17^.

Bismuth (Bi) has emerged as a top choice for heavy metal sensor construction, thanks to its excellent peak separation, low toxicity, low background current, broad potential window, and alloy-forming ability with metals like Zn, Pb, and Cd^18,19^. Bismuth oxide (Bi_2_O_3_) nanoparticles are eco-friendly and non-toxic. The reaction between heavy metals and reduced bismuth creates an “amalgam” effect, which forms an alloy-like state and enhances the surface deposition of heavy metal, thereby improving sensitivity^20,21^. However, nanoparticles tend to cluster, which limits their effectiveness in applications. To boost sensitivity in heavy metal ion detection, it’s essential to develop bismuth nanomaterials with a stable structure, controlled shape, and even dispersion. Combining Bi_2_O_3_ with carrier materials that have a high surface area, good conductivity, and chemical stability can greatly enhance its electrochemical properties^22–24^. Recently, CeO_2_ nanoparticles have gained attention for their catalytic potential in electrochemical devices, due to redox properties linked to surface defects and oxygen vacancies. Nanoscale CeO_2_ significantly increases surface area and reveals more defects, enhancing its electrochemical performance for sensor applications^25–27^.

Traditional methods for producing nanomaterials typically require a variety of hazardous chemicals, such as hydrazine, sulfuric acid, hydrofluoric acid, and hydrochloric acid^28^. These substances are generally unsafe for routine laboratory practices, leading to potential environmental and health risks. In contrast, green synthesis methods not only minimize toxic waste generation but also enhance the biocompatibility and practical usability of electrodes in environmental monitoring and sensing applications. Moreover, these eco-friendly approaches contribute significantly to sustainability by reducing chemical hazards and energy consumption in real-world applications^29–32^. This research introduces an innovative, rapid, and sustainable approach for creating a binary Bi_2_O_3_-CeO_2_ nanocomposite. A serine-assisted sol-gel auto-combustion method was applied, marking the first time this technique has been used for fabricating a Bi_2_O_3_-CeO_2_ sample. Serine molecules, with their large structures and functional groups, play a dual role in the process, acting as both an eco-friendly fuel and a structure-directing agent^33,34^. The study also developed a novel electrochemical sensor using a CeO_2_/Bi_2_O_3_-modified screen-printed electrode (CeO_2_/Bi_2_O_3_/SPE) for the simultaneous quantification of Pb(II) and Cd(II) concentrations in water and food samples. This is the first report to describe such a miniaturized CeO_2_/Bi_2_O_3_-based sensor for quantifying heavy metals. The sensor’s performance was comprehensively evaluated under optimized conditions, demonstrating excellent linearity, low detection limits (LOD), minimal interference, strong repeatability, and effective performance with real samples for detecting Pb(II) and Cd(II) ions.

## Experimental

### Chemicals and reagents

All analytical-grade reagents were utilized in this study, eliminating the need for additional purification. Deionized water was employed to prepare all solutions.

For electrochemical measurements, a PGSTAT204 electrochemical workstation (Autolab) was used. A DropSens screen-printed electrode (SPE, 110) facilitated the voltammetric analysis. Carbon was used as the material for both the working and auxiliary electrodes, while a silver or silver/silver chloride electrode served as the reference.

A field-emission scanning electron microscope (FESEM) (Zeiss Sigma 300) was used to investigate the morphology of Bi₂O₃-CeO₂ nanocomposite. Additionally, transmission electron microscopy (TEM) was conducted with a Philips CM30 TEM to analyze the internal morphology of the Bi₂O₃-CeO₂ nanocomposite.

### Green auto-combustion fabrication of Bi_2_O_3_-CeO_2_ nanocomposite

Analytically pure starting materials like bismuth (III) nitrate pentahydrate, serine, and cerium (III) nitrate hexahydrate were purchased from Merck Company and utilized as received for the preparation of Bi_2_O_3_-CeO_2_ nanocomposite. In this experimental work, a sol-gel auto-combustion reaction was employed to fabricate a Bi_2_O_3_-CeO_2_ nanocomposite using metal precursors (Bi and Ce) and a novel, environmentally friendly fuel and structure-directing agent (serine).

First, a solution containing 1 mmol of cerium nitrate and 0.4 mmol of bismuth nitrate was prepared in 15 ml of deionized water, which was mixed using a magnetic stirrer. Then, 5 ml of the solution containing serine (environmentally friendly fuel) was added drop by drop to the above solution. The ratio of serine to cerium was 4:1 mol. The mixture was stirred on a magnetic stirrer at 40 °C for 25 min. The residue was subsequently dried at 70 °C for 12 h and then calcined at 500 °C for 2 h, yielding the Bi₂O₃-CeO₂ nanocomposite sample.

### Preparation of CeO_2_/Bi_2_O_3_/SPE

To prepare the CeO_2_/Bi_2_O_3_ solution, 1 mg of prepared nanocomposite powder was introduced into 1 mL of deionized water. This mixture was ultrasonicated for 20 min, with continuous stirring for 5 min to ensure uniformity. The CeO_2_/Bi_2_O_3_/SPE was then prepared by applying a 10 µL drop of this solution onto the working electrode. The electrodes were then placed in an oven at 40 °C for 1 h to ensure complete drying of the coated layers.

### Real sample preparation

Black tea, rice, and drinking water were purchased from a local market in Kerman, Iran, and wastewater samples were collected from this city. 1 g rice sample was mixed with concentrated nitric acid (65%, 12 mL), followed by heating at 100 °C until complete dryness. After the gradual addition of 4 mL of H_2_O_2_ (30%), the mixture was heated on a hot plate until completely dry. Following this, the mixture was filtered after adding 10 mL of deionized water. The final volume was adjusted to 25 mL with deionized water before analysis.

For the black tea sample, 25 mL of acetic acid solution (0.1 M) and 1 g of tea powder were combined and left to settle for 30 min. The obtained mixture was then filtered, and the pH was set to 4.5 using a NaOH/HAc solution. The filtrate was then diluted to a final volume of 25 mL. For the drinking water and wastewater samples, a 0.22 μm membrane filter was used, and the pH was adjusted to 4.5 with a 1 M NaOH/HAc solution.

### Electrochemical parameters for the determination of Cd (II) and Pb (II)

SWASV was performed in 0.5 M acetate buffer solution (ABS; pH 4.5) with varying Cd(II) and Pb(II) levels. Metal deposition onto the Bi₂O₃/CeO₂/SPE occurred at − 1.2 V for 160 s under 1000 rpm stirring at ambient temperature, followed by stripping from − 0.9 to 0.4 V.

## Results and discussion

### Characterization of Bi_2_O_3_-CeO_2_

**Fig. 1:**
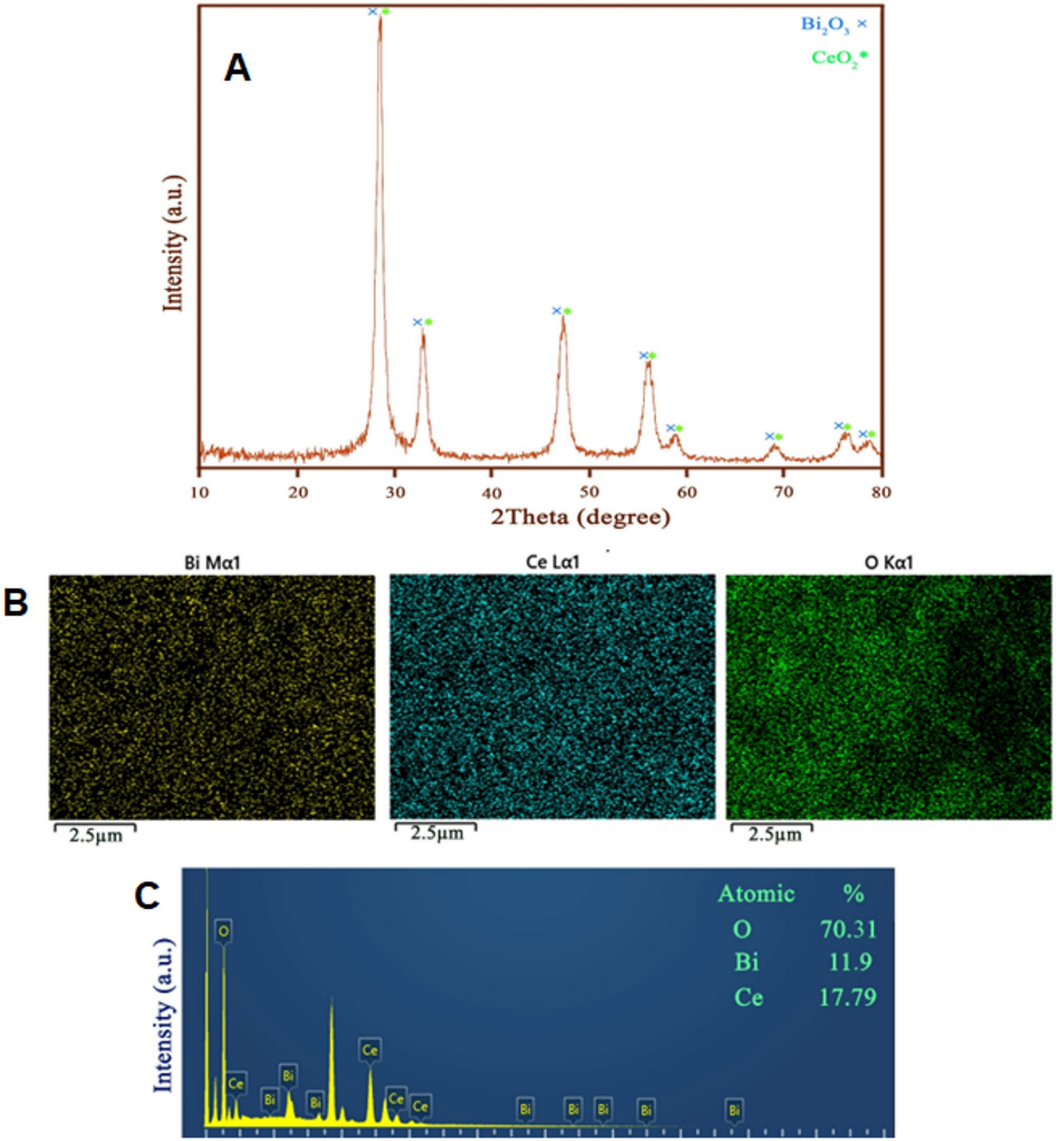
(A) XRD patterns, (B) EDS elemental mapping, and (C) EDS spectrum of the synthesized Bi_2_O_3_/CeO_2_ nanocomposite in the presence of serine.

In this research, serine molecules as a new and green fuel were applied for the easy production of Bi_2_O_3_-CeO_2_ nanocomposite samples. Figure 1A demonstrates the XRD characterizations of a sample prepared with the aid of serine as a new and environmentally friendly fuel and structure-directing agent. The signals of Bi_2_O_3_-CeO_2_ sample were observed at 2θ values 28.5214°, 32.9384°, 47.3826°, 56.1035°, 58.8410°, 69.1335°, and 76.3920° the related planes are (111), (200), (220), (311), (222), (400), and (420), correspondingly. In light of the major reflection peaks appearing in the XRD pattern, it can be understood that the produced sample includes CeO_2_ (JCPDS card 01-075-0076) and Bi_2_O_3_ (JCPDS no. 01-076-2478)^35^, both of which crystallize in the cubic phase. Thus, it can be confirmed that the cubic phase of cerium dioxide and also bismuth oxide remains the same in the synthesized binary oxide nanostructure. The appearance of sharp reflection signals in the XRD pattern of the fabricated oxide sample can be proof of its good crystallinity. To determine the mean crystal size of the Bi_2_O_3_-CeO_2_ sample synthesized with serine, Scherer’s formula was applied, and its value was about 18 nm^36^.

Figure 1B presents the elemental mapping images of the binary oxide sample prepared in the presence of serine, which was recorded with the aim of checking the chemical composition of this sample. As is evident, there is a uniform distribution of elements, including oxygen, bismuth, and cerium, on the surface of the produced oxide sample. The recorded spectrum (Fig. 1C), along with the XRD results, can confirm the successful formation of binary Bi_2_O_3_-CeO_2_ nanocomposite with the help of serine through a quick and easy approach.

**Fig. 2:**
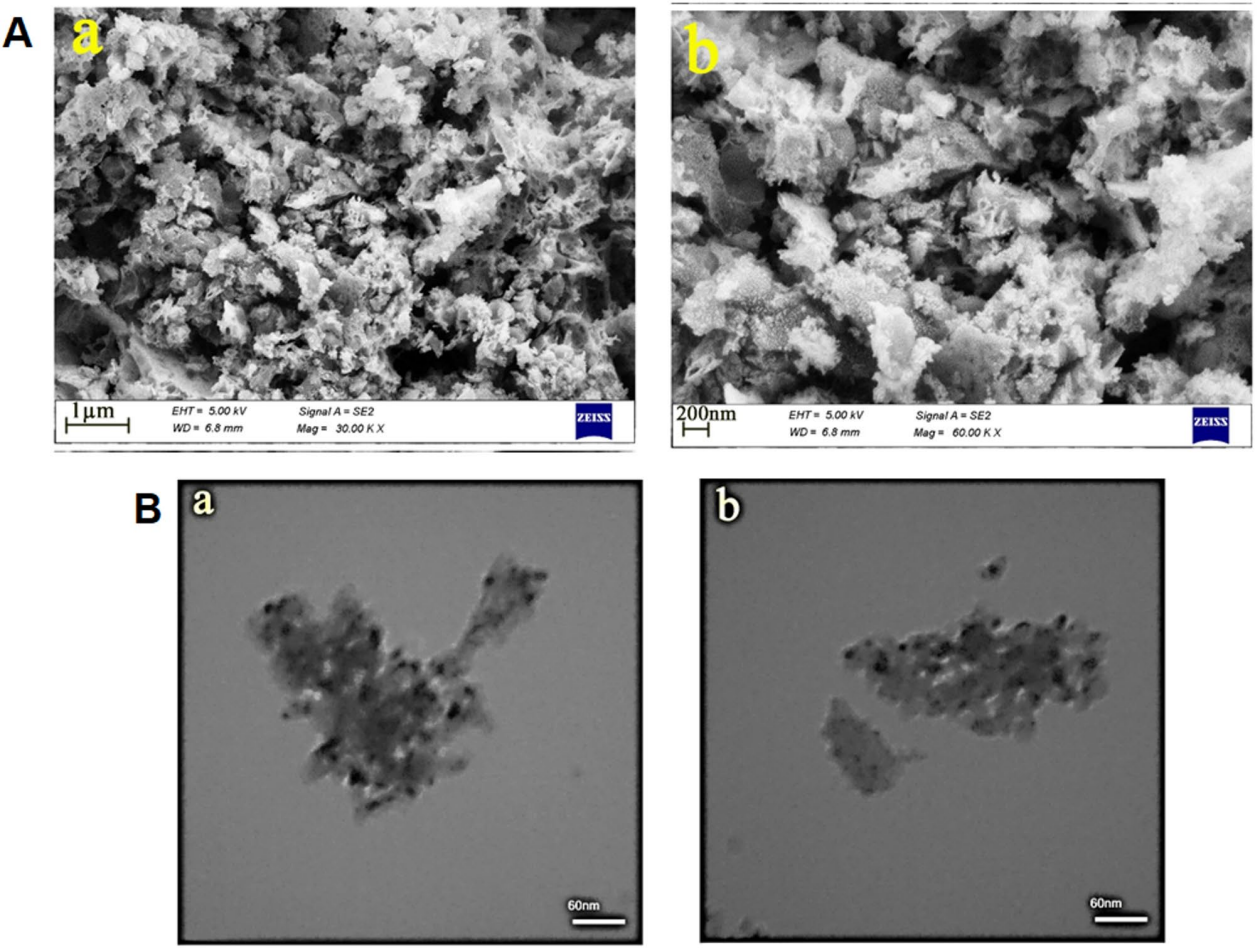
(A) Field Emission Scanning Electron Microscopy (FESEM) and (B) Transmission Electron Microscopy (TEM) images of the Bi_2_O_3_/CeO_2_ nanocomposite in different scales.

FESEM analysis was applied to examine the microstructure and morphology of the synthesized binary oxide nanostructure, as illustrated in Fig. 2A. The formation of a three-dimensional porous sponge-like nanostructure created by the assembly of nanoparticles can be seen in the images. Serine molecules, due to their large structure and functional groups, serve as both a fuel and a structure-directing agent, enabling the formation of a porous three-dimensional nanostructure^33,34^.

TEM images related to the binary Bi_2_O_3_-CeO_2_ nanocomposite produced with serine are shown in Fig. 2B. The nanostructure resulting from the assembly of nanoparticles can be seen in the images. These outcomes are in good agreement with the FESEM images of the as-prepared binary oxide sample.

### Electrochemical characteristics of CeO_2_/Bi_2_O_3_/SPE

**Fig. 3:**
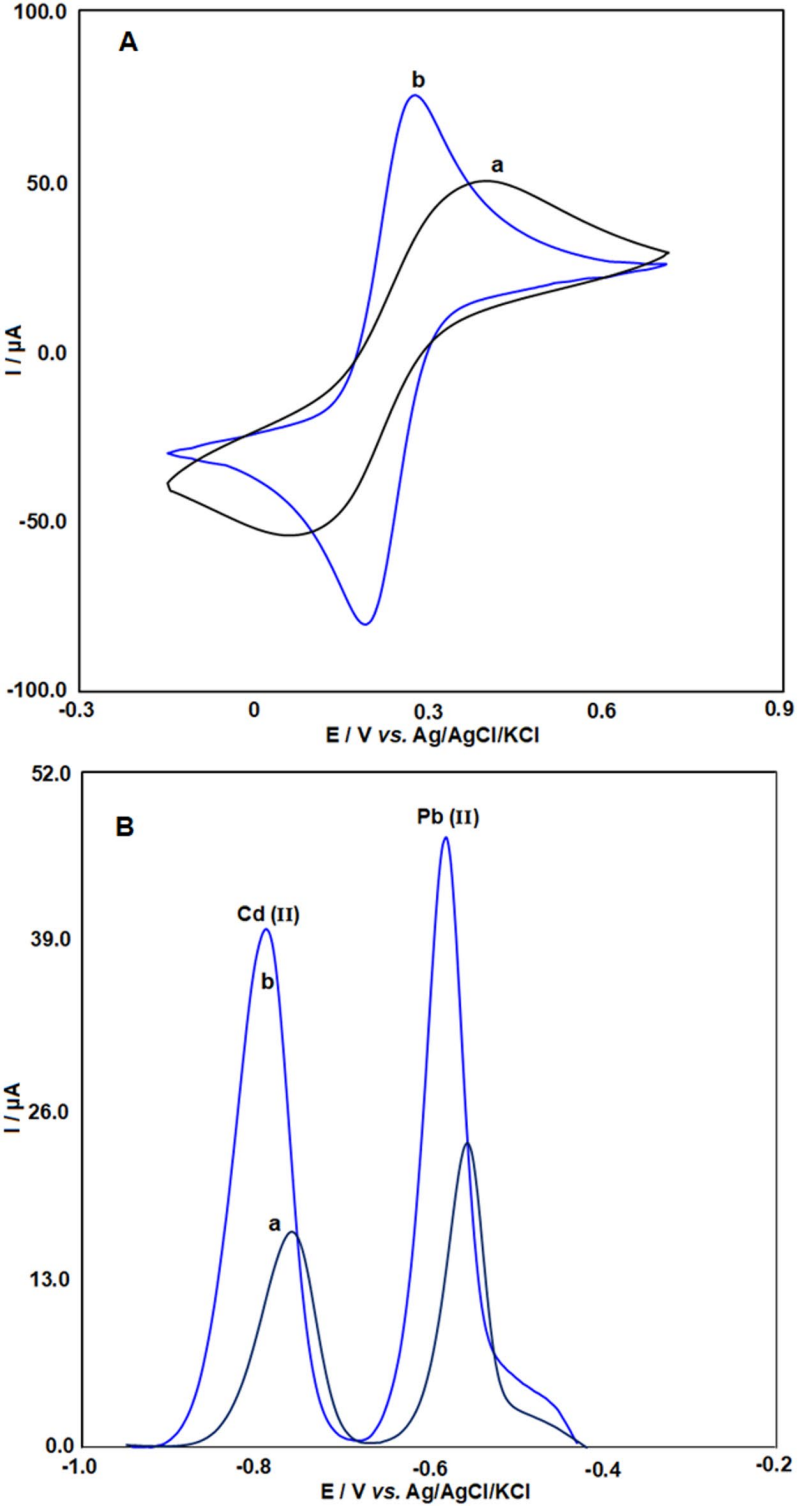
A) Cyclic voltammograms of 0.5 mM K_3_[Fe(CN)_6_] obtained with (a) bare SPE and (b) Bi_2_O_3_/CeO_2_/SPE. B) Square Wave Anodic Stripping Voltammetry results for 45 µg/L of Cd(II) and Pb(II) in ABS (0.1 M, pH 4.5) recorded on (a) bare SPE and (b) Bi_2_O_3_/CeO_2_/SPE. Deposition potential: -1.2 V; Deposition time: 160 s.

The voltammetric behavior of both bare and Bi_2_O_3_/CeO_2_/SPE was examined using a 0.5 mM redox couple with 0.1 M KCl. As shown in Fig. 3A, the Bi_2_O_3_/CeO_2_/SPE exhibited better current sensitivity compared to the unmodified SPE. The separation between the anodic and cathodic response (ΔE_p_) for the modified electrode was 84 mV, significantly lower than the value of 298 mV observed for the bare SPE. This smaller ΔE_p_ value and the increased redox current for the modified electrode suggest that the incorporation of Bi_2_O_3_/CeO_2_ facilitates faster electron transfer, which in turn enhances the electrode’s overall electrochemical performance.

To determine the effective surface area of the bare and Bi_2_O_3_/CeO_2_/SPE electrodes, the Randles–Sevcik Eq. (1) was applied:

1 $${\text{I}}_{{{\text{pa}}}} ~ = ~{\text{2}}.{\text{69}}~ \times ~{\text{1}}0^{{\text{5}}} {\text{n}}^{{{\text{3}}/{\text{2}}}} {\text{A C}}_{{\text{o}}} {\text{D}}^{{1/2}} v^{{1/2}}$$

In this equation I_pa_ represents the anodic peak current, “A” signifies the electroactive surface area of electrode, n represents the number of exchanged electrons (*n* = 1), D is the diffusion coefficient (7.6 × 10^–6^ cm^2^/s), C_o_ denotes the concentration of K_4_[Fe(CN)_6_], and *v* represents the scan rate (V/s). Based on these calculations, the electroactive surface areas of the SPE and Bi_2_O_3_/CeO_2_/SPE were determined to be 0.052 cm^2^ and 0.124 cm^2^, respectively. This increase in surface area for the modified electrode contributed to its improved electrochemical performance compared to the bare electrode.

Figure 3B illustrates the electrochemical responses of both bare SPE (a) and Bi_2_O_3_/CeO_2_/SPE (b) for detecting 45 µg/L of cadmium and lead ions in ABS (0.1 M, pH 4.5). For the bare SPE, the peak potentials (Ep) and anodic peak currents (Ip) were − 0.558 V and 21 µA for Pb(II) and − 0.754 V and 16.0 µA for Cd(II), respectively. In contrast, the modified electrode demonstrates significantly higher anodic peak currents (I_p_ = 41.0 µA for cadmium and 47.1 µA for lead ions) and lower peak potentials (E_p_ = -0.780 V for cadmium and − 0.583 V for lead ions) compared to the bare SPE. The enhanced electrochemical behavior of the Bi_2_O_3_/CeO_2_ nanocomposite, along with its large specific surface area, facilitates faster electron transfer and provides additional active sites, improving the Pb(II) and Cd(II) adsorption at the electrode surface.

### Investigation of factors influencing the sensor response

The efficiency of the electrode for simultaneously detecting lead and cadmium ions via the SWASV method is influenced by three main factors: the pH of the electrolytic solution, deposition potential, and deposition time.

**Fig. 4:**
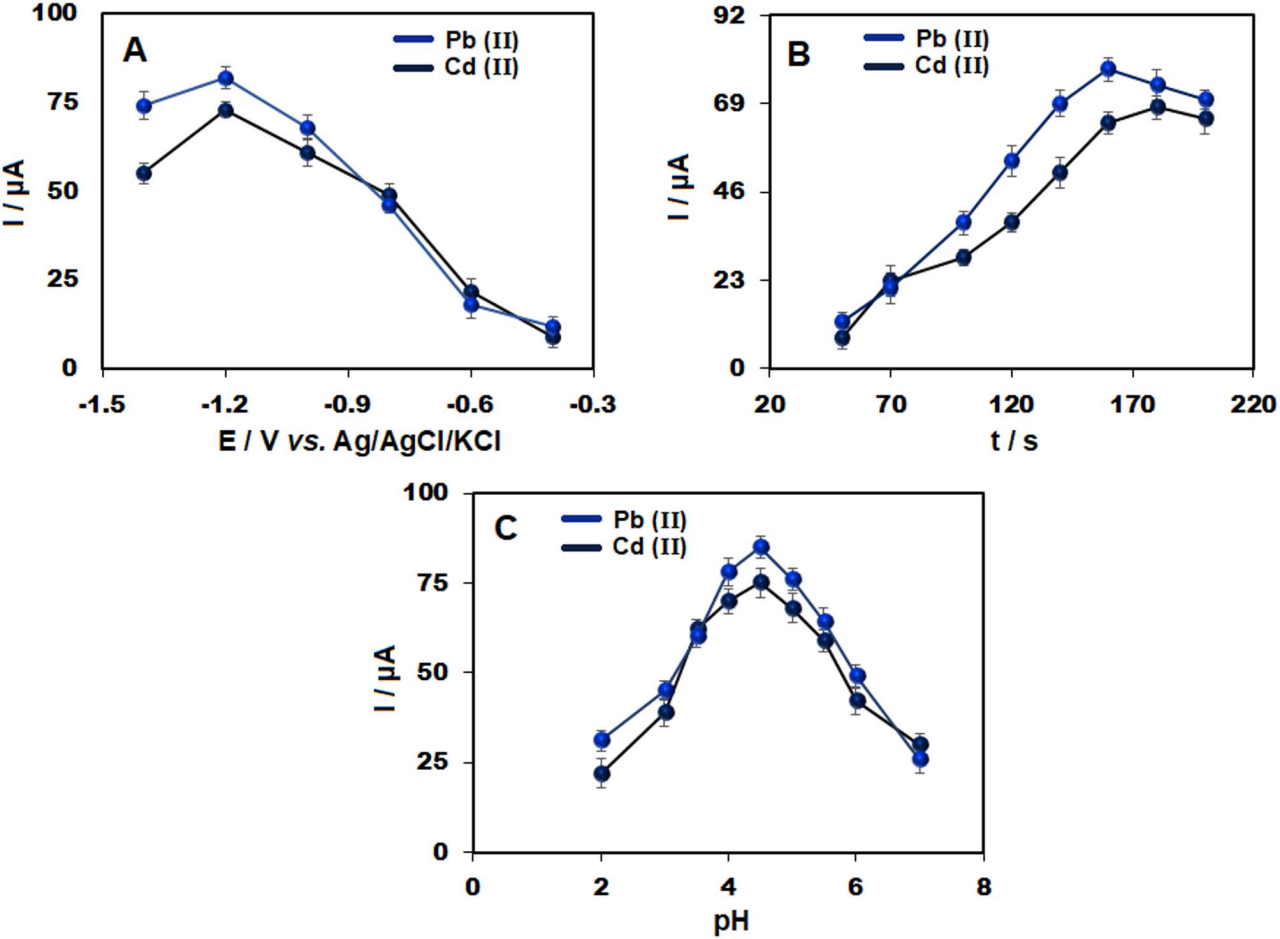
Influence of (A) deposition potential, (B) deposition time, and (C) pH on the anodic stripping currents of Cd(II) and Pb(II) (75 µg/L) to assess the performance of the Bi_2_O_3_/CeO_2_/SPE in 0.1 M ABS.

To optimize the detection, SWASV was carried out using Bi_2_O_3_/CeO_2_/SPE in ABS (0.1 M, pH 4.5), with a focus on stripping time and potential. Stripping currents increased progressively as the potential was adjusted within the range of -1.4 to -0.4 V (Fig. 4A). A more negative accumulation potential enhanced the electrochemical reduction of cadmium and lead ions, raising the peak currents. At -1.2 V, a substantial increase in current was observed, indicating a high accumulation of analytes on the modified SPE. However, when the potential was reduced to values lower than − 1.2 V, hydrogen production began to compete, inhibiting metal ion deposition on the electrode surface and resulting in a decrease in the obtained signals.

Deposition time is another crucial factor for achieving maximum sensitivity in stripping analysis. To optimize the process, SWASV was conducted using an accumulation potential set at -1.2 V in ABS (0.1 M, pH 4.5) which contains 75 µg/L of Pb(II) and Cd(II), with deposition times varied at 50, 70, 100, 120, 140, 150, 180, and 200 s. As shown in Fig. 4B, the response increased with longer deposition times but plateaued after 160 s. Thus, a time of 160 s was selected as the optimal deposition time to balance sensitivity and analysis duration.

In the present investigation, a 0.1 M ABS with a pH range of 2 to 7 was used to simultaneously detect 75 µg/L Cd(II) and Pb(II). As illustrated in Fig. 4C, pH 4.5 yielded the highest stripping peak current for both ions. Below pH 4.5, hydrogen evolution hinders heavy metal ion deposition on the electrode surface, resulting in lower peak currents. At higher pH levels, metal hydroxide complexes were formed, decreasing the metal ions concentration within the solution and resulting in lower stripping peak currents. This enhanced response at pH 4.5 can also be attributed to the favorable surface charge of the Bi₂O₃/CeO₂ nanocomposite, which promotes electrostatic attraction of Cd^2+^ and Pb^2+^ ions while maintaining efficient electron transfer kinetics. Therefore, pH 4.5 was determined to be the optimal condition for subsequent analyses.

### Application of CeO_2_/Bi_2_O_3_/SPE for determination of Cd(II) and Pb(II)

**Fig. 5:**
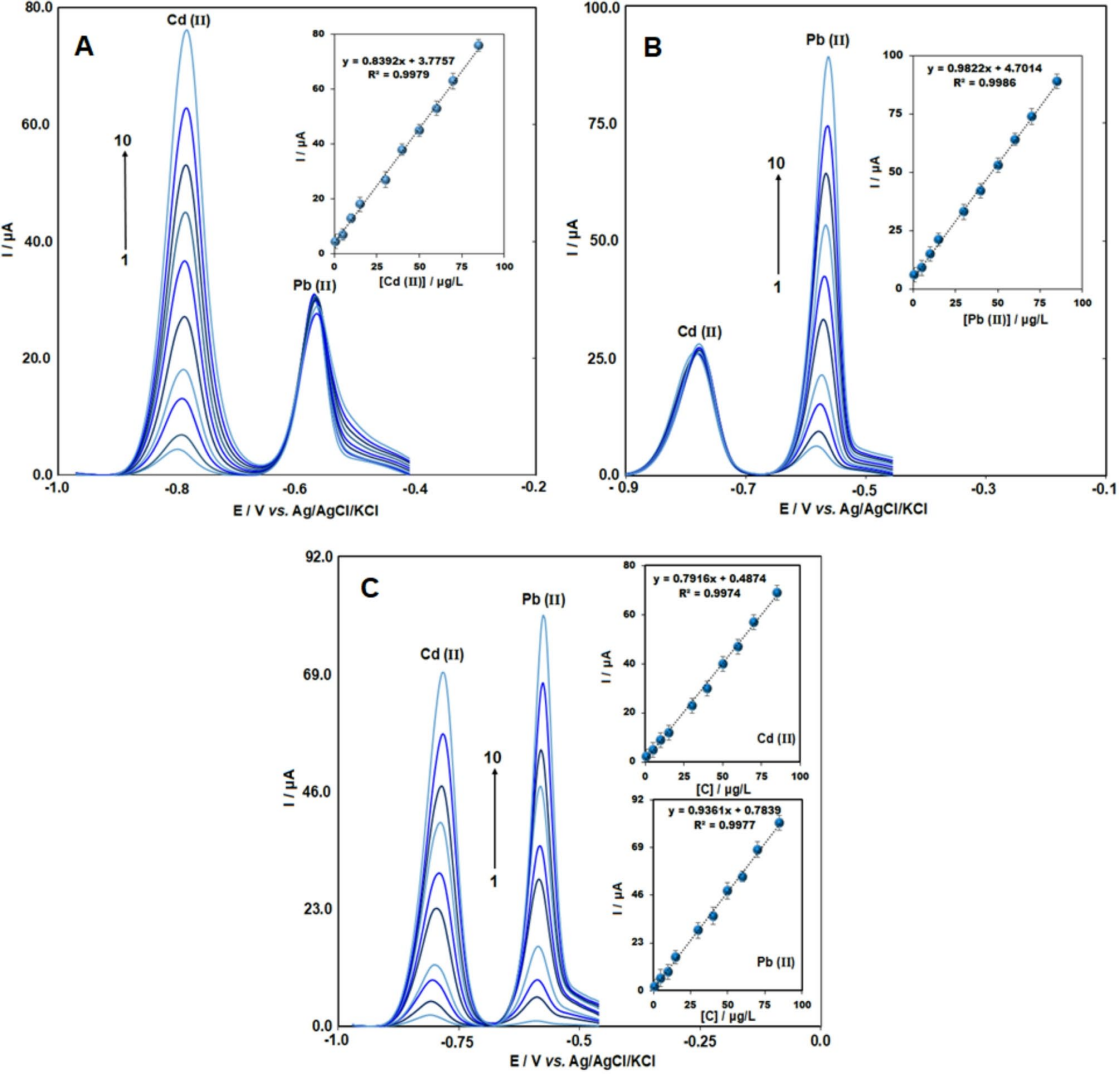
SWASV voltammograms for determining (A) Cd(II) and (B) Pb(II) (concentration range: 0.5–85 µg/L) under fixed levels of the respective other ion at the Bi_2_O_3_/CeO_2_/SPE. Insets: Calibration plots for the two ions. (C) SWASV curves for simultaneous detection of Cd(II) and Pb(II) at Bi_2_O_3_/CeO_2_ /SPE in 0.1 M ABS (pH 4.5), with concentrations ranging from 0.5 to 85 µg/L. Insets: Corresponding calibration graphs for Cd(II) and Pb(II) (*n* = 3).

To assess the impact of interference between lead and cadmium ions, SWASV analysis for simultaneous Cd(II) and Pb(II) detection was conducted by changing the concentration of one ion within the range of 0.5 to 85 µg/L while keeping the other ion fixed at 30 µg/L. Figure 5A and B show that the signals for both ions amplified linearly over the 0.5–85 µg/L concentration range, following the linear equations I_p_ = 0.83[Cd] µg/L + 3.77 (R^2^= 0.997) and I_p_ = 0.982 [Pb] µg/L + 4.7 (R^2^ = 0.998). The high correlation coefficients (R^2^) indicate a strong linear relationship for the quantification of both ions within this range. Based on the equation LOD = Sb/m, where S_b_ is the standard deviation of the blank signal and m is the slope of the calibration curve, the obtained LOD values for Cd(II) and Pb(II) were 0.14 µg/L and 0.09 µg/L, respectively. A constant or slight variation in the obtained signals was recorded for a fixed concentration of the other ion. Thus, the distinct separation of the reduction peaks for both ions ensures that the quantification of each ion does not affect the modified electrodes’ response.

The analytical responses for different concentrations of lead and cadmium ions are displayed in Fig. 5A and B, respectively. The simultaneous determination of both ions in the same solution was done using the Bi_2_O_3_/CeO_2_/SPE. Sequential additions of cadmium and lead ions at diverse concentrations were analyzed through SWVASV voltammograms, as depicted in Fig. 5C. Characteristic peaks were observed for Cd(II) and Pb(II) at -0.779 V and  -0.585 V, respectively, aligning with the expected individual responses. The concentration effects were analyzed under optimized conditions, with metal ion concentrations measured over a potential range from  -1.0 to -0.4 V and a concentration range of 0.5–85 µg/L for both ions. Minimal variation was observed in the slopes, peak positions, and calibration curves related to the individual and simultaneous determination of Pb(II) and Cd(II).

A comparison with other sensors for cadmium and lead detection (see Table 1) showed that Bi_2_O_3_/CeO_2_/SPE offered a wider linear range and a lower LOD than several other sensors. Additionally, the use of low-cost and environmentally friendly materials in constructing this electrode makes it a promising option for heavy metal analysis.

**Table 1:** Performance comparison between the proposed method and other electrochemical sensors for detecting Cd(II) and Pb(II).

Sensor	Cd^2+^ (µg/L)		Pb^2+^ (µg/L)		Refs.
	Linear range	LOD	Linear range	LOD	
Cyst/4-CP/SPCE	1.12–80	0.092	2–45	0.134	^37^
NH_2_-Ti_3_C_2_T_x_/SPE	5–100 and 100–500	0.36	10–100 and 100–500	0.31	^38^
p-DMS@MSF/ITO	30–900	2	4–1500	4	^39^
BiNPs@LPC/SPCE	0.1–150	0.03	0.1–150	0.02	^40^
Bi/GO-SPE	5 to 50	1.55	5 to 50	1.31	^41^
CS@Bi–GSPE	2.0–20.0	1.7	2.0–20.0	0.5	^42^
AuNP/0.75% APTES-ITO	5–120	0.90	5–120	0.73	^43^
[Ru(bpy)_3_]^2+^/ GO/NA/SPGE	50–350	4.2	50–350	5.3	^44^
Bi–Sb/CPE	1–200	0.27	1–150	0.29	^45^
polyPCA/GE	40 to 1000	15.4	40 to 1000	13.6	^46^
GC/GQDs-NF	20–200	11.30	20–200	8.49	^47^
Bi_2_O_3_/CeO_2_/SPE	0.5–85	0.14	0.5–85	0.09	Present study

### Stability and repeatability of CeO_2_/Bi_2_O_3_/SPE

The Bi_2_O_3_/CeO_2_/SPE electrode was tested for repeatability in measurements, demonstrating good repeatability with a relative standard deviation (RSD) of 2.9% for Pb(II) and 3.3% for Cd(II) over five consecutive measurements. The reproducibility of the Bi₂O₃/CeO₂/SPE was evaluated using five independently prepared electrodes. The RSD of the peak current responses was 3.4% for Pb(II) and 3.6% for Cd(II), indicating excellent reproducibility for both target ions.

To evaluate stability, the electrode was kept under ambient conditions at room temperature for four weeks. It retained approximately 93.2% and 93.5% of its initial current response for Pb(II) and Cd(II), respectively, indicating satisfactory durability and stability of the sensor over time.

### Effect of interfering compounds

**Fig. 6:**
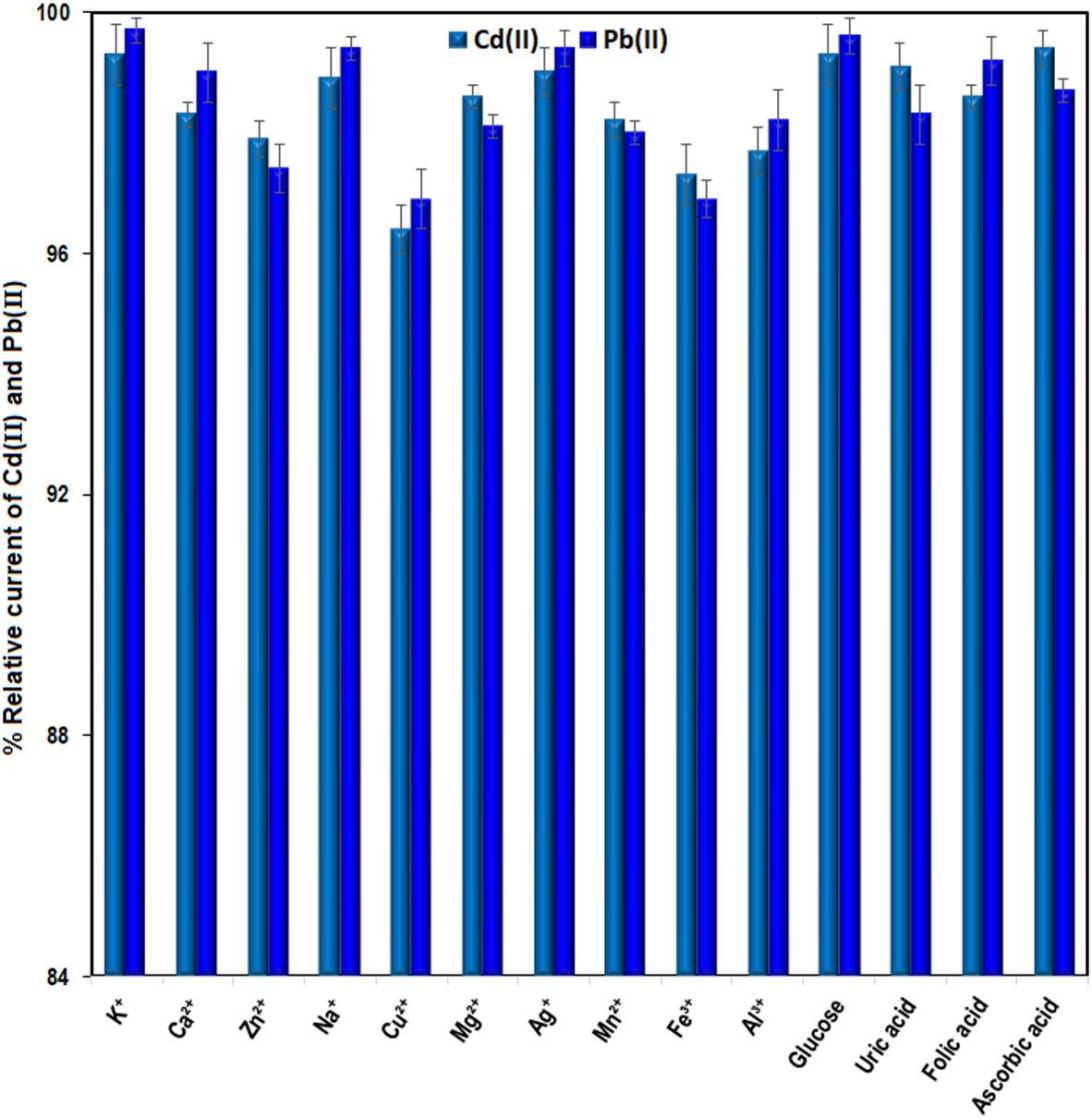
Relative current response of the Bi_2_O_3_/CeO_2_/SPE in the presence of 50.0 µg/L Cd(II) and Pb(II), along with various potential interfering substances.

To study the selectivity of the present sensor, interference tests were performed by adding a 50-fold concentration of glucose, uric acid, folic acid and ascorbic acid, a 100-fold concentration of Ag^+^, Na^+^, K^+^, Mg^2+^, Zn^2+^, Ca^2+^, Fe^3+^, Mn^2+^, and Al^3+^ ions, and a 20-fold concentration of Cu^2+^ ions, to the solution containing 50 µg/L Pb(II) and Cd(II) (see Fig. 6). The outputs showed that signal changes for lead and cadmium ions were below 5%, suggesting high selectivity with minimal interference from these ions in the electrochemical analysis of the target metals.

For higher concentrations of Cu^2+^, ferrocyanide (0.1 mM) can be used, as it leads to the formation of an insoluble copper-ferrocyanide complex, allowing for effective Cd(II) and Pb(II) determination.

### Real sample analysis

To evaluate practical reliability, the CeO_2_/Bi_2_O_3_/SPE was employed for Pb(II) and Cd(II) determination in different real samples, with outputs summarized in Table 2. Recovery rates for all ions ranged between 97% and 104%, indicating the modified electrode’s effectiveness for accurate ion determination. Additionally, the acceptable RSD values confirmed the electrode’s repeatability and precision.

**Table 2:** Analysis of Cd(II) and Pb(II) concentrations in real samples using Bi_2_O_3_/CeO_2_/SPE (*n* = 3).

Sample	Cd (II) *				Pb (II) *			
	Spiked	Found	Recovery (%)	RSD(%)	Spiked	Found	Recovery (%)	RSD(%)
Drinking Water	0.0	N.D	-	-	0.0	N.D	-	-
	8.0	7.9	98.7	2.9	7.0	7.3	104.2	3.5
	16.0	15.6	97.5	3.2	15.0	15.3	102.0	1.9
	24.0	24.8	103.3	2.4	20.0	19.6	98.0	2.6
Wastewater	0.0	35.5	-	2.5	0.0	27.6	-	3.5
	7.0	41.5	97.6	2.9	3.0	31.6	103.2	2.9
	15.0	52.3	103.5	1.9	9.0	35.8	97.8	2.9
	20.0	54.6	98.3	3.0	12.0	40.5	102.2	2.6
Black tea	0.0	3.6	-	2.9	0.0	2.9	-	3.3
	5.0	8.9	103.4	3.5	10.0	12.7	98.4	2.9
	10.0	13.9	102.2	2.4	20.0	23.6	103.0	3.1
	15.0	18.2	97.8	2.0	30.0	33.7	102.4	2.9
Rice	0.0	4.5	-	3.3	0.0	3.8	-	2.5
	10.0	15.0	103.4	3.1	5.0	8.6	97.7	2.8
	20.0	23.9	97.5	2.5	8.0	12.2	103.3	1.9
	30.0	35.3	102.3	2.7	11.0	14.5	98.0	2.5

Sample	Added Cd (II)	Found		t value	Added Pb (II)	Found		t value
		SWASV	ICP/MS			SWASV	ICP/MS	
Drinking water	0.0	N.D	N.D	-	0.0	N.D	N.D	-
	8.0	7.9 ± 0.2	8.1 ± 0.3	0.9608	15.0	15.3 ± 0.4	15.8 ± 0.3	1.7321
Black tea	0.0	3.6 ± 0.1	3.4 ± 0.2	1.5492	0.0	2.9 ± 0.1	3.2 ± 0.3	1.6432
	10.0	13.9 ± 0.3	13.7 ± 0.4	0.6928	10.0	12.7 ± 0.4	13.2 ± 0.2	1.9365

* The concentration unit for both ions in water and wastewater samples is µg/L, while for rice and tea, it is µg/Kg.

Further validation was carried out by comparing the electrochemical quantification of both ions with results obtained from using the ICP technique in two real samples. The t-test was conducted to assess the agreement between the two methods. Table 2 shows that the calculated values of t were below the critical t-value at a 95% confidence level, showing no significant difference between the outcomes of the two methods.

## Conclusion

This study presents a new eco-friendly electrochemical sensor, developed with a CeO_2_/Bi_2_O_3_ nanocomposite electrode synthesized using serine. Serine molecules acted as an innovative, environmentally sustainable fuel and structuring agent in the production of the Bi_2_O_3_/CeO_2_ nanocomposite. Through various physicochemical methods, the morphology, microstructure, and crystalline characteristics of the nanocomposite prepared with serine were thoroughly analyzed. This green synthesis approach contributes to a non-toxic sensor system. The CeO_2_/Bi_2_O_3_ modification significantly enhanced the SPE’s electrical signal, enabling effective simultaneous detection of lead and cadmium ions. The modified electrode demonstrated strong anti-interference, high repeatability, and long-term stability for heavy metal analysis. It shows great potential for integration into high-performance, reliable micro-devices designed for portable heavy metal detection applications.

## Data Availability

The datasets used and analyzed during the current study available from the corresponding author on reasonable request.
